# Genomic Assessment of Human Cumulus Cell Marker Genes as Predictors of Oocyte Developmental Competence: Impact of Various Experimental Factors

**DOI:** 10.1371/journal.pone.0040449

**Published:** 2012-07-27

**Authors:** Prisca Feuerstein, Vincent Puard, Catherine Chevalier, Raluca Teusan, Veronique Cadoret, Fabrice Guerif, Remi Houlgatte, Dominique Royere

**Affiliations:** 1 INRA, UMR85 Physiologie de la Reproduction et des Comportements, Nouzilly, France; 2 Université de Tours, Tours, France; 3 CNRS, UMR6175, Nouzilly, France; 4 CHRU de Tours, Laboratoire de Biologie de la Reproduction, Tours, France; 5 Plateforme Puces à ADN de Nantes, Institut de Recherche Thérapeutique de l’université de Nantes, Nantes, France; Hemocentro de Ribeirão Preto, HC-FMRP-USP., Brazil

## Abstract

**Background:**

Single embryo transfer (SET) is the most successful way to reduce the frequency of multiple pregnancies following *in vitro* fertilisation. However, selecting the embryo for SET with the highest chances of pregnancy remains a difficult challenge since morphological and kinetics criteria provide poor prediction of both developmental and implantation ability. Partly through the expression of specific genes, the oocyte-cumulus interaction helps the oocyte to acquire its developmental competence. Our aim was therefore to identify at the level of cumulus cells (CCs) genes related to oocyte developmental competence.

**Methodology/Principal Findings:**

197 individual CCs were collected from 106 patients undergoing an intra-cytoplasmic sperm injection procedure. Gene expression of CCs was studied using microarray according to the nuclear maturity of the oocyte (immature *vs.* mature oocyte) and to the developmental competence of the oocyte (ability to reach the blastocyst stage after fertilisation). Microarray study was followed by a meta-analysis of the behaviour of these genes in other datasets available in Gene Expression Omnibus which showed the consistency of this list of genes. Finally, 8 genes were selected according to oocyte developmental competence from the 308 differentially expressed genes (p<0.0001) for further validation by quantitative PCR (qPCR). Three of these 8 selected genes were validated as potential biomarkers (*PLIN2*, *RGS2* and *ANG*). Experimental factors such as inter-patient and qPCR series variability were then assessed using the Generalised Linear Mixed Model procedure, and only the expression level of *RGS2* was confirmed to be related to oocyte developmental competence. The link between biomarkers and pregnancy was finally evaluated and level of *RGS2* expression was also correlated with clinical pregnancy.

**Conclusion/Significance:**

*RGS2*, known as a regulator of G protein signalling, was the only gene among our 8 selected candidates biomarkers of oocyte competence to cover many factors of variability, including inter-patient factors and experimental conditions.

## Introduction

Despite its increasing use to alleviate human infertility, assisted reproductive technology (ART) continues to face two major challenges, the first being that it is relatively ineffective. The second challenge is that multiple embryo transfer has often been proposed in order to increase pregnancy rates and thus multiple pregnancies remain a common and serious complication of *in vitro* fertilisation (IVF) procedures. Moreover, the adverse outcomes associated with high-order gestations include the increased incidence of maternal, perinatal and neonatal morbidity and mortality [1]. Single embryo transfer (SET) is the most successful way to reduce the frequency of multiple pregnancies in IVF [2] but it may reduce the chance of getting pregnant. Defining the developmental competence of one oocyte after fertilisation (its ability to reach the blastocyst stage after 5/6 days of extended culture after fertilisation) and the development ability of an embryo and its implantation potential during IVF remain major goals in order to select the most suitable embryo for transfer. Morphological criteria are the most frequently used to evaluate the development potential and implantation ability of embryos in human ART. However such morphological criteria (oocyte morphology, zygote scoring, early cleavage and embryo morphology at day 2 or 3) remain poorly predictive of development or implantation ability [3]–[6]. Both genomic and proteomic analysis are difficult in human embryos, since such an approach is invasive and might affect embryo integrity [7]. Several indirect and non-invasive selection criteria focusing on oocyte or embryo quality have been proposed in the last few years.

Various studies have focused on molecules inside the follicle or the embryo microenvironment (see [8] for review). Proteomic analysis of individual human embryos [9], [10], metabolomic analysis of oocytes and embryos [11], [12] and oxygen consumption at the oocyte level [13] have all been proposed as potential biomarkers of oocyte or embryo quality.

Other studies have focused on the somatic cells (cumulus and/or granulosa cells) surrounding the oocyte since their interactions are involved in the acquisition of oocyte meiotic and developmental competence [14], [15]. Indeed specific oocyte factors are involved in the differentiation and expansion of cumulus cells (CCs) and prevent the apoptosis and luteinisation of the cumulus-oocyte complex (COC) (see [16] for review). Via such interactions, oocytes may promote specific patterns of gene expression and protein synthesis in these somatic cells [17], [18]. Several studies have therefore focused on specific gene expression in CCs according to oocyte quality in humans and animals (see [19] for review).

Developments in microarray technology have more recently allowed a global transcriptomic approach to identify differentially expressed genes according to the oocyte maturity. Studies showed different expression profiles in follicular cells according to oocyte nuclear maturity [20] or to oocyte developmental competence (early cleavage of the embryo [21], embryo quality 3 days after fertilisation [22] and implantation potential [23]).

Microarray analyses have to date focused on early embryo development (early cleavage or embryo development at day 3) or implantation ability. Early embryo development is highly dependent on oocyte quality, but embryo genome activation takes place beyond the 4 cell stage in the human [24]. Moreover, implantation involves both the development ability of the embryo and the embryo-endometrium interaction.

In an initial study, we evaluated the level of expression of 6 genes in human cumulus cells according to nuclear maturity and the developmental competence of the oocyte [25]. In this study, we undertook a global assessment of gene expression in cumulus cells. Our aim was thus to relate the transcriptome of individual human CCs to the full competence of the oocyte for pre-implantation development of the embryo as assessed by blastocyst stage development by comparing in one hand CCs from mature oocyte to immature oocyte and CCs from mature fertilised oocyte yielding a blastocyst after 5/6 days of *in vitro* culture to CCs from mature fertilised oocyte arresting development in other hand. We then analysed the behaviour of the genes related to the oocyte competence in a dataset of transcriptome of cumulus cells available in the Gene Expression Omnibus (GEO) to determine their consistency. Following this analysis, 8 genes were selected to be validated by qPCR according to their differential expression. To evaluate fully the validity of the genes as markers of oocyte developmental competence, we investigated the impact of technical and biological variability such as qPCR series and patients on the level of gene expression. Finally, the gene selected according to such criteria was investigated as a marker of pregnancy outcome. All these requirements are needed before any potential use of biomarkers to predict embryo developmental ability and finally choose the embryo for transfer.

## Materials and Methods

### Patient Selection and IVF Treatment

**Figure 1:**
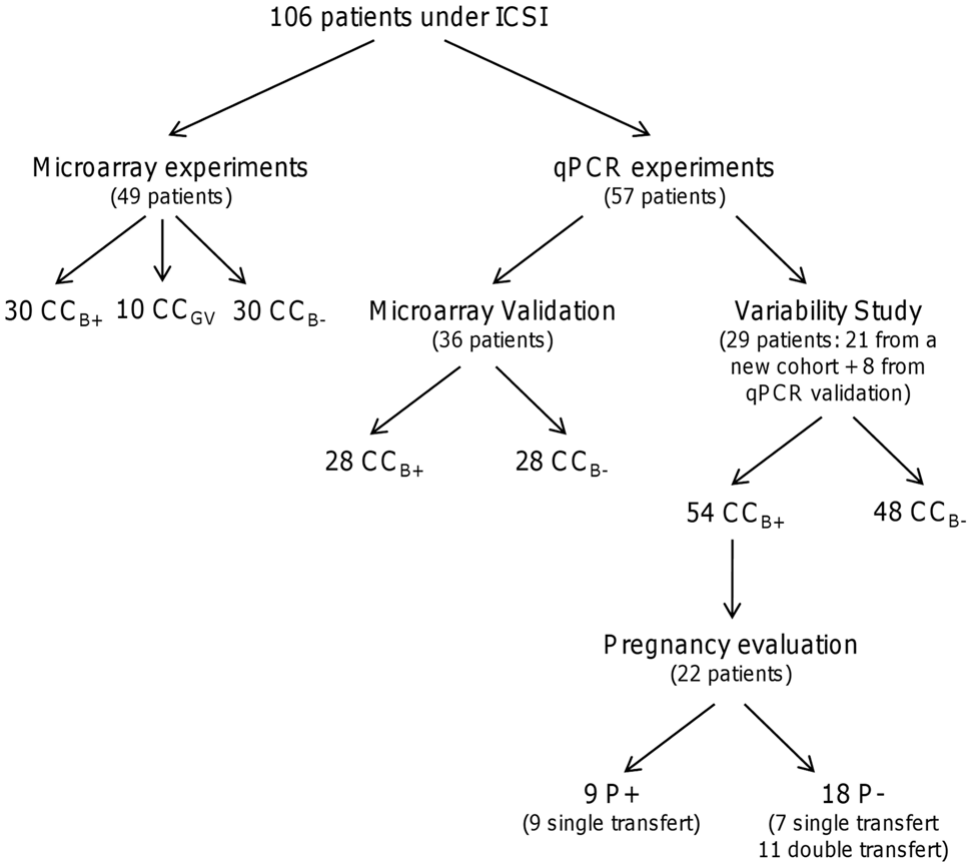
Distribution of patients included in study. Patients were separated into two main groups: microarray and qPCR. The variability group was composed of patients who had one CC_B+_ and at least one CC_B-_. The pregnancy group was composed of CC_B+_ transferred from patients included in the variability group. CC_B+_, cumulus cells from a mature oocyte yielding a blastocyst at day 5/6 of *in vitro* culture once fertilised; CC_B-_, cumulus cells from mature oocyte which stopped developing at the embryo stage at day 5/6 of *in vitro* culture once fertilised; CC_GV_, cumulus cells from immature oocyte at germinal vesicle stage; P+, pregnancy; P-, no pregnancy.

One hundred and six patients were included in this study, all undergoing an intracytoplasmic sperm injection (ICSI) procedure for male infertility. The mean number of oocytes retrieved per patient was 7 (range 3–15 oocytes). Average patient age was 33 years (range 21–42 years), 49 patients were included in the microarray analysis and 36 patients in the qPCR analysis. To further analyse variability between patients, 29 patients (21 new patients and 8 patients from qPCR analysis) were selected on the basis that at least one embryo had reached the blastocyst stage and that there was at least one arrested embryo after 6 days of extended culture. The patient groups are presented in Figure 1. The ovarian stimulation protocol, the ICSI and the embryo culture procedures have been described by Guerif *et al.* 2003 [26].

### Cumulus Cell Recovery and Assessment of Oocyte and Embryo Quality

Shortly before ICSI, individual COC were subjected to dissociation, as already described by Feuerstein *et al.* 2007 [25]. CCs were washed in cold phosphate buffer saline (80 IU/ml, SynVitro Hyadase, Medicult, Jyllinge, Denmark) then centrifuged at 300 g for 5 minutes. The supernatant was removed and the pellet was resuspended in 50 µl of RLT buffer of the RNeasy^®^ Micro Kit (Qiagen, Courtaboeuf, France) before storage at -80°C until RNA extraction. Labelling allowed individual follow-up of the whole process.

Follow-up of the morphological characteristics of the oocyte and embryo were recorded on an individual basis. Assessment of oocyte nuclear maturity and embryo quality has been described by Feuerstein *et al.* 2007 [25]. At the time of ICSI, the oocytes were first classified into two categories on the basis of nuclear status: mature oocyte with first polar body (metaphase II, MII) or immature oocyte at the germinal vesicle (GV) stage. CCs from a mature oocyte were denominated CC_MII_ and CCs from an immature oocyte CC_GV_. For mature and fertilised oocytes, we evaluated the developmental competence of each oocyte according to its ability to reach the blastocyst stage after extended culture (5 or 6 days after ICSI). As described by Feuerstein *et al.* 2007 [25], the blastocyst assessment score was based on the expansion of the blastocoel cavity and the number and cohesiveness of the inner cell mass and trophectodermal cells [27]. MII COC were divided retrospectively into two groups following the ICSI procedure on this basis, CCs from an oocyte yielding a blastocyst after 5/6 days of *in vitro* culture being denominated CC_B+_ and CCs from an oocyte arresting development at the embryo stage after 5/6 days of *in vitro* culture being denominated CC_B-_.

Clinical pregnancy was defined as described by Guerif *et al.* 2007 [4], i.e. presence of a gestational sac with a foetal heartbeat on ultrasound examination at 7 weeks of pregnancy, and the implantation rate was defined as the number of gestational sacs per number of embryos transferred. CCs from an oocyte yielding a blastocyst after 5/6 days of *in vitro* culture resulting in a clinical pregnancy were denominated P+ and CCs from an oocyte yielding a blastocyst after 5/6 days of *in vitro* culture which did not lead to a clinical pregnancy were denominated P-.

### Microarray Procedure

#### RNA extraction

Total RNA extraction and removal of genomic DNA were performed using the RNeasy^®^ Micro Kit (Qiagen, Courtaboeuf, France) according to the manufacturer’s recommendations. The quality and integrity of RNA samples used for microarray analysis were assessed using the 2100 Bioanalyser and RNA 6000 Nano LabChip kit series II (Agilent Technologies). Total RNA was quantified using a Nanodrop^®^ ND-1000 spectrophotometer (Nyxor Biotech, Paris, France). The mean quantity of RNA per cumulus was 217 ng (± 134 ng).

#### Microarray design

Ninety-six hybridisations were performed with 10 CCs from immature oocytes (GV) and 60 CCs from mature oocytes (MII), including 30 CC_B+_ and 30 CC_B-_. As far as nuclear maturity was concerned, hybridisations from 10 CCs (GV) were compared to 60 CCs (MII). Regarding developmental competence, hybridisations from 30 CC_B+_ were compared to 30 CC_B-_ , all issued from the 60 previous ones. Complementary RNA samples were prepared according to the manufacturer’s protocol (Two-Color Microarray-Based Gene Expression Analysis) and hybridised on Whole Human Genome Oligo Microarray 4x44K (Agilent Technologies). Each array contained 45,220 probes, corresponding to 41,000 single human transcripts. Briefly, an average of 72.6 ng of extracted RNA for each sample (range 65.5–89.9 ng) was amplified with one round of amplification. Each sample was labelled with cyanine 3 or cyanine 5. After purification using the RNeasy^®^ Micro Kit (Qiagen, Courtaboeuf, France), the quantity of cRNA and the specific activity of the cyanine were assessed using a Nanodrop^®^ ND-1000 spectrophotometer. Two samples (825 ng of cRNA for each) were hybridised on each slot of the 4x44K array, one sample labelled with cyanine 3 and one sample labelled with cyanine 5. In order to validate the microarray, some samples were labelled alternatively by cyanine 3 or 5, some samples were repeatedly introduced in each microarray experiment (3 experiments). After 17 hours of hybridisation, arrays were washed and scanned using the Agilent Microarray Scanner. Finally results were extracted using Feature Extraction software 9.5.1 (Agilent Technologies).

#### Microarray analysis

All quality controls were performed according to the manufacturer’s recommendations.

Lowess fitness regression was applied for global normalisation of raw expression ratios [28]. Gene expression profiles were used to classify genes, and biological samples were classified by a hierarchical analysis method using Cluster software [29], and the results of hierarchical clustering analysis were visualised using the TreeView programme. A Student t-test was applied to determine the differentially expressed genes, with a statistical significance threshold of p<0.0001. Annotations of genes and functions were performed using GoMiner software (http://discover.nci.nih.gov/gominer). Following the functional annotation of the genes, we calculated the enrichment of differentially expressed genes for each function [30]. Functions with >1.6 fold enrichment and p-value<0.001 were considered as statistically regulated according to the situation studied. The findings are accessible on the Gene Expression Omnibus (GEO) through the series accession number GSE37277.

### Meta-analysis

Datasets were obtained from the GEO (http://www.ncbi.nlm.nih.gov/geo/) and are presented in Table 1. In each dataset, probes for the 308 genes differentially expressed between CC_B-_ and CC_B+_ were investigated using MADGene [31]. Findings corresponding to these probes were extracted from each dataset. They were subjected to hierarchical clustering after log transformation and median centering of probes. The measurement used was the distance of correlation, and the aggregation method was the average linkage. The ability of these genes to discriminate samples was measured by analyzing the composition of the main separation on the sample dendrogram. Significance was calculated by Fisher’s exact test.

**Table 1:** Dataset of transcriptome of cumulus cells used for meta-analysis.

Study	Species	GEO Accession Number
Influence of hCG on the transcriptome of CCs	Mouse	GSE4260 [17]
Comparison of transcriptome of mural granulosa cells with CCs transcriptome	Human	GSE18559 [33]
Comparison of transcriptome of CCs from immature oocyte to CCs from mature oocyte	Bovine	GSE21005 [34]
CCs transcriptome according to embryo cleavage	Human	GSE9526 [21]

CCs, cumulus cells; GV, germinal vesicle stage; MII, metaphase II.

### Quantitative PCR Experiments

The following procedures were used in order to comply as far as possible with the Minimum Information for Publication of Quantitative PCR experiments MIQE guidelines.

#### RNA extraction and cDNA synthesis

Total RNA extraction and genomic DNA removal were performed as already described in microarray procedure. The quality and integrity of RNA samples were further evaluated using the RNA 6000 Pico LabChip kit series II (Agilent Technologies, Massy, France). Only RNA samples that displayed a RIN (RNA integrity number) greater than or equal to 7 were reverse transcribed to cDNA. The mean quantity of RNA per cumulus was 99 ng (range 21–205 ng). Total RNA from each sample was reverse transcribed into cDNA using the iScript^TM^ cDNA Synthesis kit (Bio-Rad Laboratories, Marnes-la-Coquette, France) with a blend of oligo(dT) and random hexamer primers to provide complete RNA sequence representation.

#### Quantitative PCR design

Samples used for the qPCR validation stage were independent of samples used for microarray hybridisations. CCs from a total of 56 mature oocytes were analysed for the qPCR validation stage, including 28 oocytes yielding a blastocyst at day 5/6 once fertilised (CC_B+_) and 28 oocytes arresting development at the embryo stage at day 5/6 once fertilised (CC_B-_).

To study the impact of patient variability and qPCR series on the level of gene expression, CCs from 102 mature oocytes were analysed, including 54 CC_B+_ and 48 CC_B-_, of which 9 CC_B+_ and 8 CC_B-_ were from the cohort of the qPCR validation stage.

To study the relationship between the level of gene expression and pregnancy, 22 patients were selected from the previous cohort from variability study (Fig. 1). Only the CC_B+_ samples were included, corresponding to 9 clinical pregnancies after transfer of a single embryo, 18 pregnancy failures represented by 7 failures after single embryo transfer and 11 after double embryo transfer.

#### Quantitative PCR

Quantitative PCR was performed using a Light Cycler apparatus with the iQ detection system and the iQ^TM^ SYBR^®^ Green Supermix kit (Bio-Rad Laboratories). Each reaction mixture contained 10 µl 2x of iQ SYBR Green Supermix (dNTPs, iTaq DNA polymerase, 6 mM MgCl_2_, SYBR Green I, fluorescein, and stabilizers), 5 µl cDNA (25-fold, 125-fold or 250-fold dilution), 300 nM of each primer and 4.5 µl of RNase free water to a final volume of 20 µl. Amplification was performed in triplicate in 96 well plates (ABgene Ltd, Epson, UK) with the following thermal cycling conditions: initial activation at 95°C for 3 minutes, followed by 40 cycles of 30 s at 95°C, 30 s at 60°C and 30 s at 72°C. A no template control (NTC) was included in all plates. Dissociation analysis of PCR products was performed by using a melting curve to confirm the absence of contaminants or primer dimers. Four-fold serial dilutions of cDNA derived from pooled human cumulus cells were used to establish the standard curve and repeated for each run as described by Feuerstein *et al.* 2007 [25].

#### Primer design

Primers were designed using the Beacon Designer version 2.0 software (Bio-Rad Laboratories) to have a melting temperature of 60°C, and if possible to cross an exon-exon junction to avoid amplification of genomic DNA. Primers used for qPCR experiments and qPCR parameters are listed in Table 2.

**Table 2:** qPCR primer sequences, PCR efficiency, correlation coefficient of standard curves, Cq range of standard curves, amplicon size and melting temperature.

GeneID	Forward and Reverse primer (5′ -3′)	E (%)	r^2^	Cq range	Tm (°C)	Product size (bp)	GeneBank No.
*ACPP*	F: TTGGAATGTTGAGAGTGTGGTTACGR: GCAGAGTGGGCAGTTTCAGC	99.3±2.5	0.995±0.002	23.9–33.9	82.5	126	NM_001099
*ANG*	F: CGAGCCACAGCGGGGTTCR: ACAGCAGAGCCAGCACTTGAC	103.1±11.8	0.994±0.003	27.8–34.4 *	87	125	NM_001097577
*ANKRD22*	F: GTGTATGTGTGTGGGCTTAGAGATTCR: TGGTATGCTGGTAAACGAACTTTATGG	101.1±16.2	0.991±0.006	28.5–35.2 *	81.5	187	NM_144590
*C10orf10*	F: GCAGCAAGAAGGTGAGGCATCR: GAGCAAGGAGGTGGCAGAGAC	96.0±6	0.993±0.002	24.1–35.3	88	142	NM_007021
*IMPA2*	F: AGCAGGCGGCATCGTGATAGR: CCAGGAGCAGAGCGTGAGC	108.9±2.1	0.995±0.003	27.7–33.8 *	91.5	256	NM_014214
*PLIN2*	F: GACAAGAGCAGCCAGGAGACCR: AGAGCAGACACCAGTTTCTACCC	92.6±0.07	0.992±0.011	20.4–31.2	84.5	400	NM_001122
*PTX3*	F: GTGTGGGTGGTGGCTTTGATGR: ATGTGGCTGGATCTCTGTGACTC	100.3±6.6	0.995±0.002	24.2–34.4	85	170	NM_002852
*RGS2*	F: CAGAACGCAAGAAGGGAATAGGTGR: TTTGGCACTCATAACGGACACTG	100.1±10.3	0.991±0.006	24.3–31 *	83	392	NM_002923
*RNF122*	F: GTAAGGTTAGAAGGGAAGGAAGGAAAGR: ACTGAGGAAGAGACTGAATGGATAGC	100.3±5.9	0.990±0.005	30.6–37.1 *	88	225	NM_024787
*RPL19*	F: TGAGACCAATGAAATCGCCAATGCR: ATGGACCGTCACAGGCTTGC	97.5±2.5	0.997±0.002	20.3–30.6	86	94	NM_000981

E, qPCR Efficiencies (mean ± SEM); r^2^, correlation coefficient of standard curves (mean ± SEM); Cq, quantification cycle; Tm, melting temperature; *, Standard curve calculated with 3 points.

#### Data analysis

Data were normalized to *RPL19* selected by the GeNorm algorithm [32] as the most stable gene. The qPCR data were recorded with iCycler IQ software version 3.1 (Bio-Rad laboratories). Melting temperatures, mean efficiency values and mean r^2^ values for standard curves are presented in Table 2. Outlier replicates of the triplicates with a variation greater than 1 quantification cycle (Cq) were excluded from the data analysis. For each sample, detection was normalized for the mean of each triplicate to *RPL19*. Each gene amplification for the qPCR validation step was performed with 3 series and 7 series for the variability study.

Statistical analysis of qPCR results was performed on 26 data points (after the deletion of outliers corresponding to the maximum and the minimum values in each group) using variance analysis followed by post-hoc comparison using the Scheffé test (Statview 4.1®, Abacus Concept, Berkeley, USA) with statistical significance defined as p<0.05.

In order to evaluate the impact of developmental competence, the patient variability and the qPCR series respectively on the level of gene expression, an analysis of variance (Anova) with the Generalised Linear Mixed Model (GLMM) procedure was performed for each gene with Statistical Analysis System (SAS®) software. The model chosen was fitted for each gene independently *y_ijkn_* = *μ*+*P_i_*+*A_j_*+*Q_k_*+*P_i_A_j_*+*ε_ijkn_*, where *y_ijkn_* is the gene expression level in the *n*th CC for a gene according to the *i*th phenotype from the *j*th patient after the *k*th qPCR series, *μ* is the gene expression level mean, P*i* the fixed effect of the *i*th phenotype (*i* =  CC_B+_ or CC_B-_), A*j* the random effect of the *j*th patient (*j* = 1 to 29), Q*k* the random effect of the *k*th qPCR series (*k* = 1 to 7), (PA)*ij* the first-order interaction between variables phenotype and patient, and ε_ijkn_ the residual random effect. The levels of significance of the model and the different effects were set at p<0.01 and p<0.05, respectively.

The relationship between the level of expression of candidate genes and pregnancy was assessed using one way analysis of variance, Bartlett’s test to compare variances, followed by post-hoc comparison using the Scheffé test (p<0.05).

## Results

### Microarray Analysis

**Figure 2:**
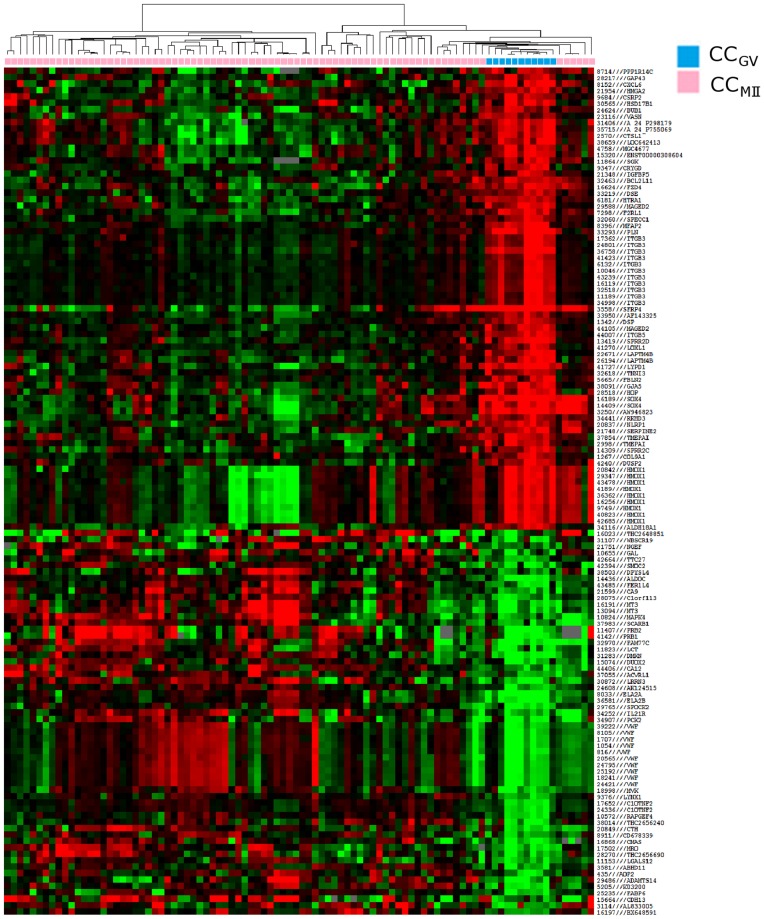
Heat map and cluster dendograms of gene clusters differentially expressed according to oocyte nuclear maturity. Hierarchical clustering of cumulus cell samples (columns) and the 132 most significant probes (rows). Upregulated genes are marked in red, downregulated genes are marked in green. CC_GV_ (blue), cumulus cells from immature oocyte at germinal vesicle stage; CC_MII_ (pink), cumulus cells from mature oocyte.

Initially, differentially expressed genes were analysed according to the nuclear maturity of the oocyte. From the 45,220 probe sets in the array, 15,531 unique genes were expressed in cumulus cells, among which 724 unique genes (854 probes) were differentially expressed between CC_GV_ and CC_MII_. Six hundred and thirty-four genes were upregulated and 90 genes were downregulated in CC_MII_ compared to CC_GV_. Hierarchical clustering based on the 132 most differentially expressed genes allowed separation of all CC_GV_ from other samples, corresponding to CC_MII_ (Fig. 2). Sixteen functions were upregulated in CC_MII_ as compared to CC_GV,_ according to the calculated enrichment and p-value of each function (Table 3). Among them the following annotations should be emphasized: activation of MAPKK activity, positive regulation of lipid biosynthesis process, caspase activator activity, caspase regulator activity and apoptotic protease activator activity. Following similar criteria, 37 functions were downregulated in CC_MII_ compared to CC_GV_ (Table 4), among which the following annotated functions should be emphasized: tRNA processing, induction of apoptosis, induction of programmed cell death, tRNA metabolisis.

**Table 3:** Upregulated functions in CC_MII_ compared to CC_GV_.

Function	p-value
oxidoreductase activity	0.0011
lipid biosynthetic process	0.0015
caspase regulator activity	0.0018
phosphatidylcholine transmembrane transporter activity	0.0019
caspase activator activity	0.0038
endoplasmic reticulum	0.0042
activation of MAPKK activity	0.0054
neuron migration	0.0054
positive regulation of lipid biosynthesis process	0.0054
organic acid biosynthesis process	0.0065
carboxylic acid biosynthesis process	0.0065
receptor signaling protein serine/threonine kinase activity	0.0074
apoptotic protease activator activity	0.0077
regulation of caspase activity	0.0085
regulation of peptidase activity	0.0085
regulation of endopeptidase activity	0.0085

CC_MII_, cumulus cells from mature oocyte; CC_GV_, cumulus cells from oocyte at germinal vesicle stage.

**Table 4:** Downregulated functions in CC_MII_ compared to CC_GV_.

Function	p-value
vitamin binding	0.0021
tRNA processing	0.0036
positive regulation of development process	0.0068
4-aminobutyrate transaminase activity	0.0071
N-acetyl-gamma-glutamyl-phosphate reductase activity	0.0071
phosphoglucomutase activity	0.0071
pseudouridylate synthase activity	0.0071
vitamin D binding	0.0071
bleomycin hydrolase activity	0.0071
queuine tRNA-ribosyltransferase activity	0.0071
queuosine biosynthesisprocess	0.0071
7-methylguanosine metabolism process	0.0071
nucleoside biosynthesis process	0.0071
response to tropane	0.0071
tyrosyl-DNA phosphodiesterase activity	0.0071
stem cell maintenance	0.0071
calcium channel inhibitor activity	0.0071
pyridoxal phosphate binding	0.0071
troponin T binding	0.0071
4-aminobutyrate transaminase complex	0.0071
succinate-semialdehyde dehydrogenase binding	0.0071
response to cocaine	0.0071
purine nucleoside biosynthesis process	0.0071
ribonucleoside biosynthesis process	0.0071
MCM complex	0.0071
positive regulation of axon extension	0.0071
guanosine biosynthetic process	0.0071
queuosine metabolic process	0.0071
7-methylguanosine biosynthesis process	0.0071
purine ribonucleoside biosynthesis process	0.0071
behavioral response to cocaine	0.0071
stem cell differentiation	0.0071
stem cell development	0.0071
cofactor binding	0.0078
induction of apoptosis	0.0091
induction of programmed cell death	0.0093
tRNA metabolism process	0.0094

CC_MII_, cumulus cells from mature oocyte;

CC_GV_, cumulus cells from oocyte at germinal vesicle stage.

**Figure 3:**
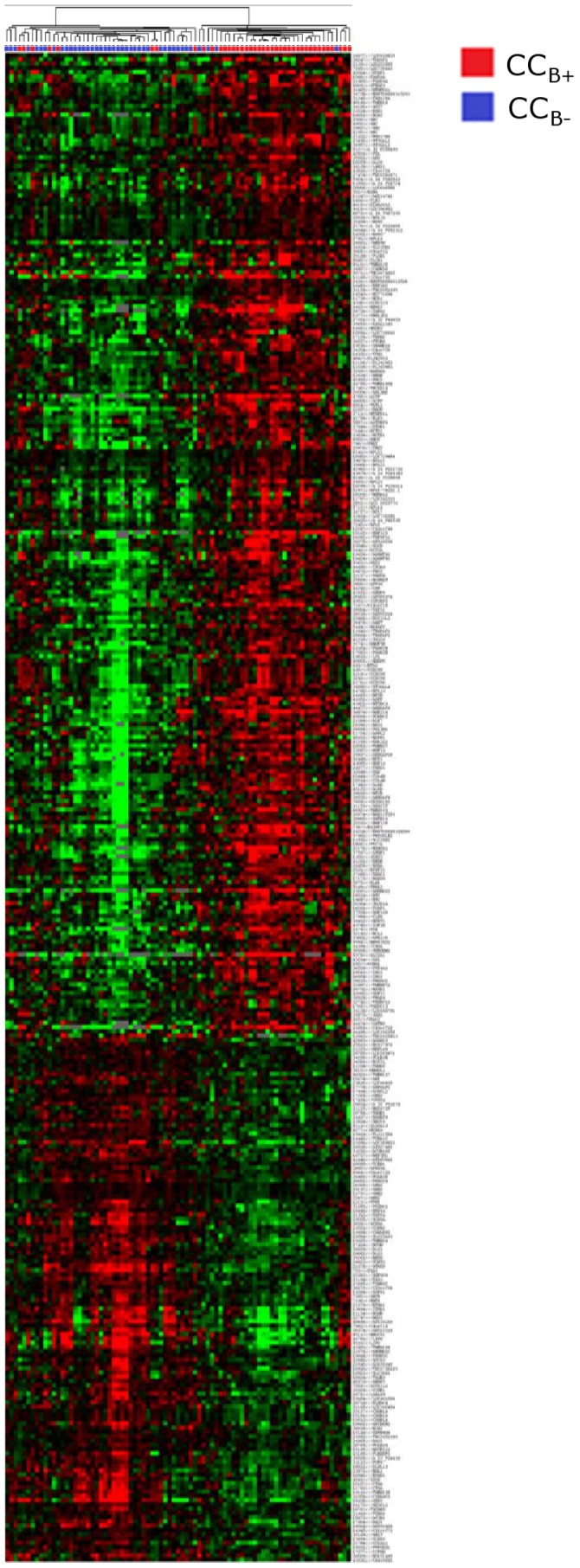
Heat map and cluster dendograms of gene clusters differentially expressed according to oocyte developmental competence. Hierarchical clustering of cumulus cell samples (columns) and the 354 most significant probes (rows). Upregulated genes are marked in red, downregulated genes are marked in green. CC_B-_ (blue), cumulus cells from mature oocyte which arrested at the embryo stage at day 5/6 of *in vitro* culture once fertilised; CC_B+_ (red), cumulus cells from mature oocyte yielding a blastocyst at day 5/6 of *in vitro* culture once fertilised.

Differentially expressed genes were then analysed according to the ability of the oocyte to yield a blastocyst. From the 45,220 probes set on the array, 354 were differentially expressed between CC_B+_ and CC_B-_. These 354 probes referred to 308 single genes, with 133 genes downregulated and 175 genes upregulated in CCs enclosing a mature oocyte yielding a blastocyst, compared to those unable to reach this stage. The hierarchical clustering based on the 354 differentially expressed probes allowed separation of almost all CC_B+_ from CC_B-_ (Fig. 3). Upregulation of 23 functions was observed in CC_B+_ compared to CC_B-_, including negative regulation of cell differentiation, fatty acid biosynthesis, organic acid biosynthesis, carboxylic acid biosynthesis and transcription factor binding (Table 5). On the other hand, 31 functions such as cell redox homeostasis, cyclin-dependent protein kinase regulator activity, respiratory gaseous exchange and transporter activity were downregulated in CC_B+_ (Table 6).

**Table 5:** Upregulated functions in CC_B+_ compared to CC_B-_.

Function	p-value
cytosolic large ribosomal subunit (sensu Eukaryota)	0.0006
zinc ion binding	0.0015
cation binding	0.0019
negative regulation of cell differentiation	0.0030
fatty acid biosynthesis	0.0040
negative regulation of myeloid cell differentiation	0.0045
cytosolic ribosome (sensu Eukaryota)	0.0048
organic acid biosynthesis	0.0060
carboxylic acid biosynthesis	0.0060
ribosome biogenesis and assembly	0.0063
large ribosomal subunit	0.0064
ion binding	0.0069
metal ion binding	0.0069
mesonephros development	0.0073
phosphocreatine metabolism	0.0073
porphobilinogen synthase activity	0.0073
10-formyltetrahydrofolate biosynthesis	0.0073
creatine kinase activity	0.0073
depyrimidination	0.0073
depurination	0.0073
male germ-line stem cell division	0.0073
transcription factor binding	0.0093
transcription regulator activity	0.0099

CC_B+_, cumulus cells from mature oocyte yielding a blastocyst at day 5/6 of *in vitro* culture once fertilised;

CC_B-_, cumulus cells from mature oocyte which stopped developing at the embryo stage at day 5/6 of *in vitro* culture once fertilised.

**Table 6:** Downregulated functions in CC_B+_ compared to CC_B-_.

Function	p-value
cell redox homeostasis	0.0022
cyclin-dependent protein kinase regulator activity	0.0025
transmembrane receptor protein serine/threonine kinase signalling pathway	0.0026
respiratory gaseous exchange	0.0048
thiamin diphosphokinase activity	0.0048
Etioplast	0.0048
regulation of border follicle cell delamination	0.0048
border follicle cell delamination	0.0048
thiamin diphosphate biosynthesis	0.0048
thiamin diphosphate metabolism	0.0048
septate junction assembly	0.0048
establishment and/or maintenance of neuroblast polarity	0.0048
asymmetric protein localization during cell fate commitment	0.0048
positive regulation of developmental growth	0.0048
regulation of developmental growth	0.0048
G1/S transition checkpoint	0.0048
alpha(1.6)-fucosyltransferase activity	0.0048
glycoprotein 6-alpha-L-fucosyltransferase activity	0.0048
transporter activity	0.0060
hydroxymethylglutaryl-CoA synthase activity	0.0095
GARP complex	0.0095
thiamin and derivative biosynthesis	0.0095
establishment and/or maintenance of polarity of larval imaginal disc epithelium	0.0095
bisphosphoglycerate phosphatase activity	0.0095
bisphosphoglycerate mutase activity	0.0095
zonula adherens assembly	0.0095
basal protein localization	0.0095
collagen type VI	0.0095
phospholipase A1 activity	0.0095
rhythmic excitation	0.0095
outward rectifier potassium channel activity	0.0095

CC_B+_, cumulus cells from mature oocyte yielding a blastocyst at day 5/6 of *in vitro* culture once fertilised;

CC_B-_, cumulus cells from mature oocyte which stopped developing at the embryo stage at day 5/6 of *in vitro* culture once fertilised.

### Meta-analysis

We further analysed the behavior of our 308 genes discriminating CC_B-_ and CC_B+_ in other datasets available in the GEO [17], [21], [33], [34]. All the probes corresponding to the 308 genes using MADGene [31] were extracted in each study. Data from these probes were log-transformed and median centered and subjected to hierarchical classification. The ability of these probes to discriminate sample types was measured by Fisher’s exact test on sample composition of the main separation on the sample dendrogram.

**Figure 4:**
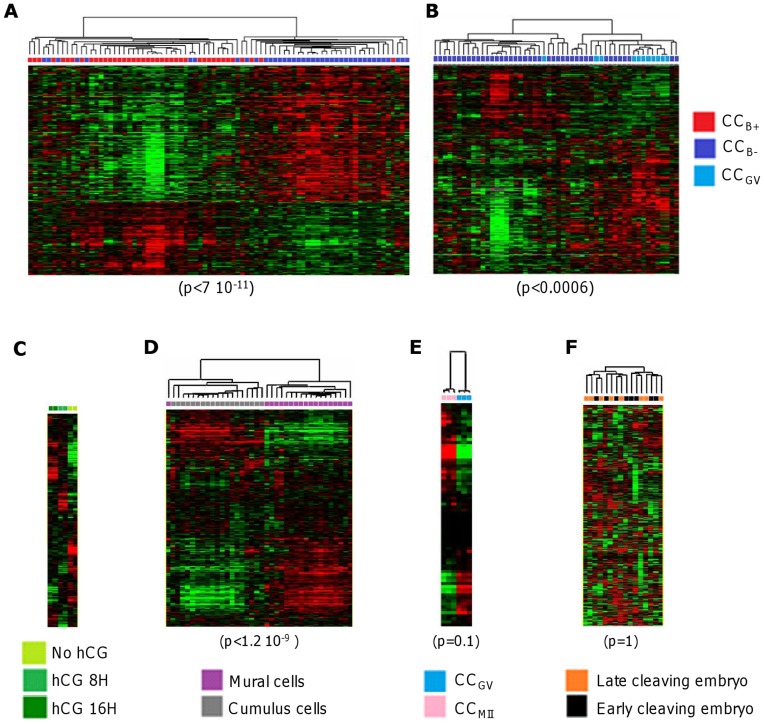
Hierarchical clustering of microarray datasets. Measurements of the 308 genes discriminating CC_B-_ and CC_B+_ were extracted from different datasets: our study (A, B), GSE4260 (C), GSE18559 (D), GSE21005 (E) and GSE9526 (F). They were subjected to hierarchical clustering after log transformation and median centering of genes. The different types of sample are shown as coloured squares. The quality of the separation was measured by Fisher’s exact test on the main branch. CC_GV_, cumulus cells from immature oocyte at germinal vesicle stage; CC_MII_, cumulus cells from mature oocyte; CC_B+,_ cumulus cells from mature oocyte yielding a blastocyst at day 5/6 of *in vitro* culture once fertilised; CC_B-,_ cumulus cells from mature oocyte which stopped developing at the embryo stage at day 5/6 of *in vitro* culture once fertilised.

The results are shown in Figure 4. The 308 genes allowing separation of almost all CC_B+_ from CC_B-_ (Fig. 4A) were under the influence of hCG (Fig. 4C). These genes allowed discrimination of mural granulosa cells from CCs, as expected of cumulus genes (Fig. 4D). Moreover, these genes seemed to discriminate the degree of nuclear maturity of the oocytes although without statistical significance (Fig. 4E) while they allowed separation of almost all CC_B-_ from CC_GV_ in our study (Fig 4B). However, they did not discriminate the developmental stage of the embryo (early *vs.* late cleaving embryo) (Fig. 4F). These results strongly support the fact that our 308 genes expressed in CCs were presented in the datasets previously reported and their expression well correlated with the described conditions.

### qPCR Validation

#### Comparison between microarray and qPCR

Another set of samples was used for qPCR validation.

**Figure 5:**
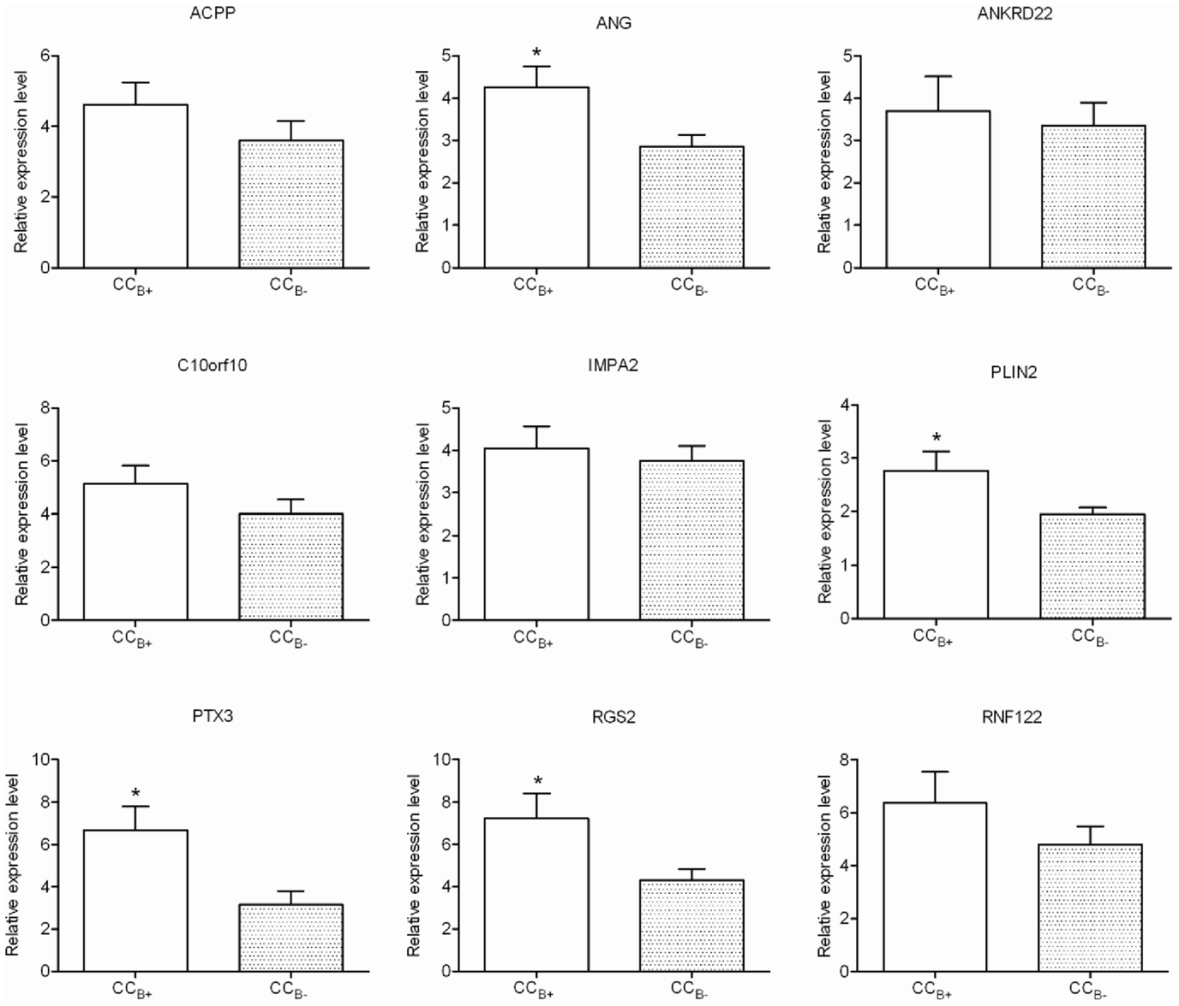
Relative expression level obtained by qPCR of 9 genes differentially expressed according to oocyte developmental competence. Results were expressed as means ± SEM of relative expression to the reference gene RPL19. CC_B+,_ cumulus cells from mature oocyte yielding a blastocyst at day 5/6 of *in vitro* culture once fertilised; CC_B-,_ cumulus cells from mature oocyte which stopped developing at the embryo stage at day 5/6 of *in vitro* culture once fertilised; *, significant difference (p<0.05).

Eight genes that were upregulated in CC_B+_ as compared to CC_B-_ [*ACPP (acid phosphatase prostate), ANG (angiogenin), ANKRD22 (ankyrin repeat domain 22), C10orf10 (chromosome 10 open reading frame 10,* also known as *DEPP or FIG), IMPA2 (inositol(myo)-1(or-4)-monophosphatase 2), PLIN2 (perilipin 2), RGS2 (regulator of G-protein signalling 2)* and *RNF122 (ring finger protein 122)*] on the microarray study were selected for qPCR validation. The 8 selected genes revealed the same expression profile in qPCR as in microarray, with statistical significance for three (*PLIN2, RGS2* and *ANG*) (Fig. 5).

#### Assessment of the respective impact of patients and qPCR series on the expression level of ANG, PLIN2 and RGS2

In order to further validate *ANG*, *PLIN2* and *RGS2* as potential biomarkers of oocyte developmental competence, we assessed the impact of patients and of qPCR series on the level of gene expression according to oocyte developmental competence. Using a Generalised Linear Mixed Model, we found the statistical significance of the model for the 3 genes. Except for *PLIN2*, gene expression was influenced by the qPCR series (Table 7). Moreover, the levels of expression of *ANG* and *RGS2* were subjected to a patient effect. Finally, the level of expression of *RGS2* remained clearly related to oocyte developmental competence, while taking into account the influence of patient and qPCR series without any interaction between patient and phenotype.

### Further Validation Stage of RGS2: Link to Pregnancy

Having assessed the validity of *RGS2* expression, we then tested the hypothesis of a relationship between implantation and the level of expression of this gene. Of the 22 patients who had a blastocyst transfer in the previous cohort, 9 became pregnant (only after single blastocyst transfer) whereas 13 did not (7 single blastocyst transfers and 11 double blastocyst transfers). The level of expression (mean ± SEM) of *RGS2* was significantly increased in the pregnant compared to the non-pregnant group (4.77±1.68 *vs.* 1.75±0.23, p<0.05).

### Comparison of RGS2 to a Known Potential Biomarker: PTX3

In addition we compared the relevance of our new potential biomarker *RGS2* to a known potential biomarker (Pentraxin 3, *PTX3*). *RGS2* presented significantly increased expression in CC_B+_ as compared to CC_B-_ on microarray and qPCR analysis. In the microarray analysis, the expression of *PTX3* was slightly upregulated in CC_B+_ compared to CC_B-_ (fold change = 1.21, p = 0.12). In qPCR analysis, the level of expression of *PTX3* was significantly higher in CC_B+_ than in CC_B-_ (6.44±5.71 *vs.* 3.04±3.39, respectively) (Fig. 5). Finally, as with the level of expression of *RGS2,* that of *PTX3* was significantly increased in the pregnant group compared to the non-pregnant group (7.01±3.64 *vs.* 1.25±0.34). However, a significant interaction between patient and phenotype factors was observed for *PTX3* expression level (Table 7).

## Discussion

Following global genomic assessment of the human cumulus cell transcriptome, 724 genes were found to be differentially expressed according to the nuclear maturity of the oocyte and 308 other genes according to the developmental competence of the oocyte. These two series had no genes in common. Comparison of our list of 308 genes with other datasets of CCs transcriptomes showed that these genes were both under the influence of hCG, and they discriminated mural granulosa cells from CCs. They also demonstrated the degree of nuclear maturity of the oocytes as in our study but were independent with regard to the early embryo development at day 2/3. With qPCR experiments on 8 selected genes, we validated 3 of them as potential biomarkers *ANG*, *PLIN2* and *RGS2*. After further validation, *RGS2* seemed to be the most pertinent biomarker since its expression was correlated both with oocyte developmental competence, despite the patient variability, and with the clinical pregnancy.

Non-invasive assessment of embryo quality remains a major goal, since the contribution of morphological evaluation of early embryo development to prediction of further development or implantation remains quite limited [4]. Among the various “omic” approaches to the environment of the oocyte or the embryo, studies on somatic cells in close contact with the oocyte represent one alternative (see [35] for review). Indeed, the CCs surrounding the oocyte contribute substantially to oocyte growth and maturation. Gene expression at the level of CCs may reflect essential stages in oocyte/cumulus interactions during oocyte maturation and thus offers an indirect non-invasive way to assess oocyte competence.

Studies in humans have to date focused either on the developmental competence of the embryo (early cleavage at day 1 [21]; embryo quality at day 2 or 3 [22], [36]–[38]) or on the implantation ability of the embryo [22], [23], [38], [39]. Embryonic genome activation in the human occurs between the 4- and 8-cell stages [24]. However, reaching the 8-cell stage does not guarantee further development to the blastocyst stage. Moreover, the implantation ability of the embryo also depends on uterine receptivity. Thus, as this parameter might create bias and lead to overlooking of crucial factors for embryo quality, we chose to relate the developmental competence of the oocyte at day 5/6 after ICSI to gene expression at the cumulus level before assessing the ability to implant. Individual retrieval of CCs and individual follow-up of oocyte maturity and embryo development and pregnancy outcome allowed us to relate each cumulus expression profile to the maturity of the oocyte of origin, subsequent embryo quality and pregnancy outcome.

Our strategy was based on four stages: 1) transcriptomic approach followed by a meta-analysis from other datasets of the CCs transcriptome to validate the consistency of our list of genes; 2) validation of potential biomarkers using qPCR in relation to oocyte competence; 3) analysis of variability linked to patient and/or qPCR series; and 4) evaluation of the predictive value of biomarkers of clinical pregnancy. Three independent sets of samples were used for the different stages of this study (microarray and qPCR experiments). Indeed, Van Montfort *et al.* have shown the importance of using independent samples for validation to ensure that the expression profile was really correlated with the situations being compared and was not due to the samples [21].

**Table 7:** Test of hypotheses for Mixed Model Analysis of Variance, impact of developmental competence, patient variability and experience (*i.e.* qPCR series) on the level of gene expression.

Effects / yijkn (Gene)	P*i (Phenotype)*	A*j (Patient)*	Q*k (qPCR series)*	(PA)*ij (Patient* * *Phenotype)*
*ANG*	0.1353	<0.0001*	0.0044*	1.00
*PLIN2*	0.3113	0.6204	0.4347	0.0550
*RGS2*	0.0201*	0.0014*	0.0168*	0.1880
*PTX3*	0.0991	0.9196	0.0229*	<0.0001*

*y_ijkn_* gene expression level in *n*th cumulus cells for a gene according to the *i*th phenotype from *j*th patient after *k*th qPCR series; *μ* gene expression level mean; P*i* fixed effect of *i*th phenotype (*i* =  CC_B+_ cumulus cells from mature oocyte yielding a blastocyst at day 5/6 of *in vitro* culture once fertilised and *i* =  CC_B-_ cumulus cells from mature oocyte which stopped developing at the embryo stage at day 5/6 of *in vitro* culture once fertilised); A*j* random effect of the *j*th patient (*j* = 1 to 29); Q*k* random effect of *k*th qPCR series (*k* = 1 to 7); (PA)*ij* first-order interaction between variables phenotype and patient;

* Statistical significance of model factors for the levels of expression of the four genes (p<0.05).

At this point the considerable heterogeneity between the potential biomarkers reported in the literature should be noted (see [40], [41] for reviews). However such heterogeneity might at least partly be explained by methodological differences. For example, RNA extraction conditions, RNA sample amplification depending on priming conditions, hybridisation sample labelling, reverse transcriptase source, and finally level of significance for gene selection may all have an impact on the genes listed [42]. O’Shea *et al.* showed the importance of cross comparison of datasets to identify biomarkers [43]. We therefore choose to perform a meta-analysis by comparing our own list of genes in terms of oocyte developmental competence to other datasets of CCs from the mouse [17], humans [21], [33] and bovines [34] with regard to hCG influence, the difference in transcriptome between mural granulosa cells and CCs, the degree of oocyte maturity and early embryo development, respectively. Despite such heterogeneity, the results demonstrated a certain consistency over the different conditions of these studies. Interestingly, these 308 genes separated almost all the CCs according to oocyte maturity in our study. However, the power of separation was weaker than for the 724 genes that we identified in our study. The meta-analysis strongly supported the fact that the 308 genes were consistent with results of published datasets.

One major problem may occur with the variability encountered between the patients themselves and the experimental procedures. Few reports have focused on these aspects, which are very important if the use of such evaluations is to be promoted prospectively, where the independence of the biomarker from the patient and the experiment is essential [38], [44]. Hamel *et al.* reported a very interesting way of studying intra-patient variability, considering both one or two embryos yielding pregnancy and one arrested embryo for each patient, while inter-patient experimentation focused on groups of embryos of different status (pregnancy, non-pregnancy, embryo failure) which allowed delineation of the impact of the status rather than the inter-patient variability itself [38]. Principal Component Analysis was used to discriminate true positive embryos when two transferred embryos yielded a single pregnancy. Interestingly, the same group recently reported follicular marker genes as pregnancy predictors for human IVF. While considering only the pregnancy as an endpoint rather than both development characteristics and pregnancy, they reported certain differences concerning expression levels of the same genes between the two experiments reported to date [23], [45]. Moreover, the second study emphasized the predictive value of UDP-glucose pyrophosphorylase-2 and pleckstrin homology-like domain, familyA, member1, which were not mentioned in the previous report. In addition to the difference in phenotype selection, the influence of experimental conditions cannot be excluded.

To evaluate further the respective influences of developmental competence and patient variability and qPCR series variability at the level of gene expression, we studied a set of 29 patients with at least one blastocyst and one arrested embryo per patient. Analysis was based on an Anova test with GLMM to combine the multiple factors, developmental competence as fixed variable, and patient and qPCR series as random variables. Only 1 of the 8 candidate genes (i.e. *RGS2*) remained related to oocyte developmental competence, independently of patient and qPCR series variability. We can therefore assume some congruence with the results of Hamel [38]. Interestingly, this biomarker may also be related to clinical pregnancy, since its level of expression has been shown to be significantly increased in successful transfers compared to implantation failures. As already mentioned, a recent study showed that the expression level of *RGS2* in human follicular cells might be considered as a good predictor of ongoing pregnancy [38].

The *RGS2* gene encodes a GTPase-activating protein that hydrolyses GTP to GDP on theαsubunit of an activated G-protein [46]. *Rgs2* is expressed in rat granulosa cells after hCG injection prior to the ovulation [47] and is probably involved in the regulation of granulosa cells response to gonadotrophins. Up regulation of *RGS2* by hCG in human and mouse granulosa cells was recently confirmed with the ability of that protein to block hCG induced downstream target gene COX2 trancription through the Gαs pathway [48]. Another possibility might concern a regulatory activity of RGS2 on ion channels. Recently RGS2 was reported to interact with a scaffolding protein spinophilin to regulate calcium signalling in xenopus laevis oocytes [49], [50].

If pregnancy is considered as the major endpoint, it is important to note that, having applied statistical analysis to each of the eight biomarkers investigated in our study, only one was selected, *i.e. RGS2.* In addition we compared this gene to a known potential biomarker *PTX3*. This finding is consistent with other studies which identified *RGS2* in follicular cells and *PTX3* in CCs as markers of pregnancy ([38] and [51], respectively). However, the latter biomarker cannot be kept, since it was shown to involve a significant interaction between patient and phenotype.

This study focused on 8 selected genes among 308 discriminative ones. The results presented do not exclude the presence of biomarkers among other members of this list. However, we may assume that, of all the putative candidate genes, the follicular biomarker(s) of oocyte competence or pregnancy prediction selected need to encompass all variability factors, including patient and qPCR series factors. *RGS2* was the only gene among our selected candidates to fulfil these requirements in this study. Understanding the biological role of *RGS2* during the oocyte-cumulus interaction and prospective double blind evaluation of this biomarker (and others candidates) will be the next steps. This latter condition has to concile with cost/effectiveness evaluation, since time and cost constraints as well as routinely use may differ in dedicated gene expression or eg. protein expression arrays before any embryo transfer strategy based on biomarkers assessment might be applied [52].

## References

[1] Land JA, Evers JL (2003) Risks and complications in assisted reproduction techniques: Report of an ESHRE consensus meeting. Hum Reprod 18: 455–457. 12571190 10.1093/humrep/deg081

[2] Bergh C (2005) Single embryo transfer: a mini-review. Hum Reprod 20: 323–327. 15665008 10.1093/humrep/deh744

[3] Rienzi L, Vajta G, Ubaldi F (2011) Predictive value of oocyte morphology in human IVF: a systematic review of the literature. Hum Reprod Update 17: 34–45. 20639518 10.1093/humupd/dmq029 PMC3001337

[4] Guerif F, Le Gouge A, Giraudeau B, Poindron J, Bidault R, et al (2007) Limited value of morphological assessment at days 1 and 2 to predict blastocyst development potential: A prospective study based on 4042 embryos. Hum Reprod 22: 1973–1981. 17496054 10.1093/humrep/dem100

[5] Rijnders PM, Jansen CA (1998) The predictive value of day 3 embryo morphology regarding blastocyst formation, pregnancy and implantation rate after day 5 transfer following in-vitro fertilization or intracytoplasmic sperm injection. Hum Reprod 13: 2869–2873. 9804247 10.1093/humrep/13.10.2869

[6] Milki AA, Hinckley MD, Behr B (2002) Comparison of blastocyst transfer to day 3 transfer with assisted hatching in the older patient. Fertil Steril 78: 1244–1247. 12477519 10.1016/s0015-0282(02)04273-5

[7] Jones GM, Cram DS, Song B, Kokkali G, Pantos K, et al (2008) Novel strategy with potential to identify developmentally competent IVF blastocysts. Hum Reprod 23: 1748–1759. 18477572 10.1093/humrep/den123

[8] Royere D, Feuerstein P, Cadoret V, Puard V, Uzbekova S, et al (2009) Non invasive assessment of embryo quality: proteomics, metabolomics and oocyte-cumulus dialogue. Gynecol Obstet Fertil 37: 917–920. 19836287 10.1016/j.gyobfe.2009.09.016

[9] Katz-Jaffe MG, Gardner DK (2006) Schoolcraft WB (2006) Proteomic analysis of individual human embryos to identify novel biomarkers of development and viability. Fertil Steril 85: 101–107. 16412738 10.1016/j.fertnstert.2005.09.011

[10] Katz-Jaffe MG, McReynolds S, Gardner DK (2009) Schoolcraft WB (2009) The role of proteomics in defining the human embryonic secretome. Mol Hum Reprod 15: 271–277. 19223337 10.1093/molehr/gap012 PMC2666223

[11] Singh R, Sinclair KD (2007) Metabolomics: Approaches to assessing oocyte and embryo quality. Theriogenology 68: S56–S62. 17490741 10.1016/j.theriogenology.2007.04.007

[12] Botros L, Sakkas D, Seli E (2008) Metabolomics and its application for non-invasive embryo assessment in IVF. Mol Hum Reprod 14: 679–690. 19129367 10.1093/molehr/gan066 PMC2639446

[13] Scott L, Berntsen J, Davies D, Gundersen J, Hill J, et al (2008) Human oocyte respiration-rate measurement potential to improve oocyte and embryo selection? Reprod Biomed Online 17: 461–469. 18854099 10.1016/s1472-6483(10)60232-5

[14] Canipari R (2000) Oocyte-granulosa cell interactions. Hum Reprod Update 6: 279–289. 10874573 10.1093/humupd/6.3.279

[15] Gilchrist RB, Ritter LJ, Armstrong DT (2004) Oocyte-somatic cell interactions during follicle development in mammals. Anim Reprod Sci 82–83: 431–446. 10.1016/j.anireprosci.2004.05.017 15271471

[16] Gilchrist RB, Lane M, Thompson JG (2008) Oocyte-secreted factors: regulators of cumulus cell function and oocyte quality. Hum Reprod Update 14: 159–177. 18175787 10.1093/humupd/dmm040

[17] Hernandez-Gonzalez I, Gonzalez-Robayna I, Shimada M, Wayne CM, Ochsner SA, et al (2006) Gene Expression Profiles of Cumulus Cell Oocyte Complexes during Ovulation Reveal Cumulus Cells Express Neuronal and Immune-Related Genes: Does this Expand Their Role in the Ovulation Process? Mol Endocrinol 20: 1300–1321. 16455817 10.1210/me.2005-0420

[18] Su Y-Q, Sugiura K, Wigglesworth K, O’Brien MJ, Affourtit JP, et al (2008) Oocyte regulation of metabolic cooperativity between mouse cumulus cells and oocytes: BMP15 and GDF9 control cholesterol biosynthesis in cumulus cells. Development 135: 111–121. 18045843 10.1242/dev.009068

[19] Li Q, McKenzie LJ, Matzuk MM (2008) Revisiting oocyte-somatic cell interactions: In search of novel intrafollicular predictors and regulators of oocyte developmental competence. Mol Hum Reprod. gan064. 10.1093/molehr/gan064 PMC2639448 18996952

[20] Ouandaogo ZGl, Haouzi D, Assou S, Dechaud H, Kadoch IJ, et al (2011) Human Cumulus Cells Molecular Signature in Relation to Oocyte Nuclear Maturity Stage. PLoS ONE 6: e27179 EP -. 10.1371/journal.pone.0027179 PMC3210145 22087263

[21] van Montfoort AP, Geraedts JP, Dumoulin JC, Stassen AP, Evers JL, et al (2008) Differential gene expression in cumulus cells as a prognostic indicator of embryo viability: a microarray analysis. Mol Hum Reprod 14: 157–168. 18204071 10.1093/molehr/gam088

[22] Assou S, Haouzi D, Mahmoud K, Aouacheria A, Guillemin Y, et al (2008) A non-invasive test for assessing embryo potential by gene expression profiles of human cumulus cells: a proof of concept study. Mol Hum Reprod 14: 711–719. 19028806 10.1093/molehr/gan067

[23] Hamel M, Dufort I, Robert C, Gravel C, Leveille M-C, et al (2008) Identification of differentially expressed markers in human follicular cells associated with competent oocytes. Hum Reprod 23: 1118–1127. 18310048 10.1093/humrep/den048

[24] Braude P, Bolton V, Moore S (1988) Human gene expression first occurs between the four- and eight-cell stages of preimplantation development. Nature 332: 459–461. 3352746 10.1038/332459a0

[25] Feuerstein P, Cadoret V, Dalbies-Tran R, Guerif F, Bidault R, et al (2007) Gene expression in human cumulus cells: one approach to oocyte competence. Hum Reprod 22: 3069–3077. 17951581 10.1093/humrep/dem336

[26] Guerif F, Cadoret V, Poindron J, Lansac J, Royere D (2003) Overnight incubation improves selection of frozen-thawed blastocysts for transfer: preliminary study using supernumerary embryos. Theriogenology 60: 1457–1466. 14519467 10.1016/s0093-691x(03)00130-4

[27] Gardner D (1999) Schoolcraft W (1999) In vitro culture of human blastocyst. In: Jansen R, Mortimer D, editors. Towards reproductive certainty: infertility and genetics beyond. Carnforth, UK: Parthenon Press. 378–388.

[28] Yang YH, Dudoit S, Luu P, Lin DM, Peng V, et al (2002) Normalization for cDNA microarray data: a robust composite method addressing single and multiple slide systematic variation. Nucleic Acids Res 30: e15. 11842121 10.1093/nar/30.4.e15 PMC100354

[29] Eisen MB, Spellman PT, Brown PO, Botstein D (1998) Cluster analysis and display of genome-wide expression patterns. Proc Natl Acad Sci USA 95: 14863–14868. 9843981 10.1073/pnas.95.25.14863 PMC24541

[30] Zeeberg BR, Feng W, Wang G, Wang MD, Fojo AT, et al (2003) GoMiner: a resource for biological interpretation of genomic and proteomic data. Genome Biol 4: R28. 12702209 10.1186/gb-2003-4-4-r28 PMC154579

[31] Baron D, Bihouee A, Teusan R, Dubois E, Savagner F, et al (2011) MADGene: retrieval and processing of gene identifier lists for the analysis of heterogeneous microarray datasets. Bioinformatics 27: 725–726. 21216776 10.1093/bioinformatics/btq710 PMC3042180

[32] Vandesompele J, De Preter K, Pattyn F, Poppe B, Van Roy N, et al (2002) Accurate normalization of real-time quantitative RT-PCR data by geometric averaging of multiple internal control genes. Genome Biol 3: RESEARCH0034. 12184808 10.1186/gb-2002-3-7-research0034 PMC126239

[33] Koks S, Velthut A, Sarapik A, Altmae S, Reinmaa E, et al (2010) The differential transcriptome and ontology profiles of floating and cumulus granulosa cells in stimulated human antral follicles. Mol Hum Reprod 16: 229–240. 19933312 10.1093/molehr/gap103

[34] Regassa A, Rings F, Hoelker M, Cinar U, Tholen E, et al (2011) Transcriptome dynamics and molecular cross-talk between bovine oocyte and its companion cumulus cells. BMC Genomics 12: 57. 21261964 10.1186/1471-2164-12-57 PMC3045333

[35] Fauser BC, Diedrich K, Bouchard P, Dominguez F, Matzuk M, et al (2010) Contemporary genetic technologies and female reproduction. Hum Reprod Update 17: 829–847. 10.1093/humupd/dmr033 PMC3191938 21896560

[36] Zhang X, Jafari N, Barnes RB, Confino E, Milad M, et al (2005) Studies of gene expression in human cumulus cells indicate pentraxin 3 as a possible marker for oocyte quality. Fertil Steril 83 Suppl 11169–1179. 15831290 10.1016/j.fertnstert.2004.11.030

[37] Anderson RA, Sciorio R, Kinnell H, Bayne RA, Thong KJ, et al (2009) Cumulus gene expression as a predictor of human oocyte fertilisation, embryo development and competence to establish a pregnancy. Reproduction 138: 629–637. 19602522 10.1530/REP-09-0144

[38] Hamel M, Dufort I, Robert C, Leveille M-C, Leader A, et al (2010) Genomic assessment of follicular marker genes as pregnancy predictors for human IVF. Mol Hum Reprod 16: 87–96. 19778949 10.1093/molehr/gap079

[39] Assidi M, Montag M, Van der Ven K, Sirard MA (2011) Biomarkers of human oocyte developmental competence expressed in cumulus cells before ICSI: a preliminary study. J Assist Reprod Genet 28: 173–188. 20953827 10.1007/s10815-010-9491-7 PMC3059525

[40] Assou S, Haouzi D, De Vos J, Hamamah S (2010) Human cumulus cells as biomarkers for embryo and pregnancy outcomes. Mol Hum Reprod 16: 531–538. 20435608 10.1093/molehr/gaq032

[41] Huang Z, Wells D (2010) The human oocyte and cumulus cells relationship: new insights from the cumulus cell transcriptome. Mol Hum Reprod 16: 715–725. 20435609 10.1093/molehr/gaq031

[42] Gilbert I, Scantland S, Sylvestre EL, Dufort I, Sirard MA, et al (2010) Providing a stable methodological basis for comparing transcript abundance of developing embryos using microarrays. Mol Hum Reprod 16: 601–616. 20479066 10.1093/molehr/gaq038

[43] O’Shea LC, Mehta J, Lonergan P, Hensey C, Fair T (2012) Developmental competence in oocytes and cumulus cells: candidate genes and networks. Syst Biol Reprod Med 0: 1–14. 10.3109/19396368.2012.656217 22313243

[44] Adriaenssens T, Wathlet S, Segers I, Verheyen G, De Vos A, et al (2010) Cumulus cell gene expression is associated with oocyte developmental quality and influenced by patient and treatment characteristics. Hum Reprod. deq049. 10.1093/humrep/deq049 20228394

[45] Hamel M, Dufort I, Robert C, Leveille MC, Leader A, et al (2010) Identification of follicular marker genes as pregnancy predictors for human IVF: new evidence for the involvement of luteinization process. Mol Hum Reprod 16: 548–556. 20610614 10.1093/molehr/gaq051

[46] Dohlman HG, Thorner J (1997) RGS Proteins and Signaling by Heterotrimeric G Proteins. J Biol Chem 272: 3871–3874. 9064301 10.1074/jbc.272.7.3871

[47] Ujioka T, Russell DL, Okamura H, Richards JS, Espey LL (2000) Expression of Regulator of G-Protein Signaling Protein-2 Gene in the Rat Ovary at the Time of Ovulation. Biol Reprod 63: 1513–1517. 11058559 10.1095/biolreprod63.5.1513

[48] Wu YL, Chuang HH, Kou YR, Lee TS, Lu SH, et al (2008) Regulation of LH receptor and PGF2alpha receptor signaling by the regulator of G protein signaling 2 (RGS2) in human and mouse granulosa cells. Chin J Physiol 51: 282–291. 19175184

[49] Wang X, Zeng W, Soyombo AA, Tang W, Ross EM, et al (2005) Spinophilin regulates Ca2+ signalling by binding the N-terminal domain of RGS2 and the third intracellular loop of G-protein-coupled receptors. Nat Cell Biol 7: 405–411. 15793568 10.1038/ncb1237

[50] Wang X, Zeng W, Kim MS, Allen PB, Greengard P, et al (2007) Spinophilin/neurabin reciprocally regulate signaling intensity by G protein-coupled receptors. EMBO J 26: 2768–2776. 17464283 10.1038/sj.emboj.7601701 PMC1888664

[51] Gebhardt KM, Feil DK, Dunning KR, Lane M, Russell DL (2011) Human cumulus cell gene expression as a biomarker of pregnancy outcome after single embryo transfer. Fertil Steril 96: 47–52 e42. 10.1016/j.fertnstert.2011.04.033 21575950

[52] Ferrari S, Lattuada D, Paffoni A, Brevini TA, Scarduelli C, et al (2010) Procedure for rapid oocyte selection based on quantitative analysis of cumulus cell gene expression. J Assist Reprod Genet 27: 429–434. 20467802 10.1007/s10815-010-9428-1 PMC2922707

